# A Conceptual Framework of Destination Sustainability in Sharing Economy

**DOI:** 10.1007/978-3-030-65785-7_41

**Published:** 2020-11-28

**Authors:** Huiying Zhang, Xi Yu Leung, Billy Bai

**Affiliations:** 1 Department for Informatics, Technical University of Munich, Garching bei München, Bayern Germany; 2 Smart Tourism Education Platform (STEP) College of Hotel and Tourism Management, Kyung Hee University, Seoul, Korea (Republic of); 3 Department of Tourism and Service Management, MODUL University Vienna, Vienna, Wien Austria; 4 University of Nevada, Las Vegas, Las Vegas, NV 89154 USA; 5 University of North Texas, Denton, TX 76203 USA

**Keywords:** Resident-tourist relationship, Sharing economy, Destination sustainability, Stakeholder theory, Collaboration theory, Resource theory, Service-dominant logic, Capital Theory Approach (CTA), Transformative e-Tourism

## Abstract

The introduction of the sharing economy has revolutionized resident-tourist relationships and provides further implications of destination sustainability. Built on several well-established theories, this conceptual study intends to develop a new and holistic framework to examine destination sustainability, focusing on the change of resident-tourist relationships. The framework is first guided by the stakeholder theory to identify the four key stakeholders in the new sharing economy context: residents, tourists, governments, and the sharing economy platform. With the collaboration theory and resource theory as a foundation, the framework then describes each stakeholder’s specific needs and resources. The service-dominant logic further supports service exchanges and value co-creation among stakeholders. The framework then adopts the capital theory approach to conceptualize destination sustainability in terms of human, social, natural and manufactured capital. Finally, three propositions are developed to justify the new peer-to-peer collaboration paradigm that leads to destination sustainability. The proposed framework is aligned with the six-pillar transformation in e-Tourism research and serves as an intelligent solution to destination sustainable development in the sharing economy context.

## Introduction

The interaction between tourists and residents is fundamental to tourism destinations (Sharpley 2014). However, tourists’ activities negatively impact residents’ quality of life and local sustainability (Routledge 2001), and waves of anti-tourism movements exacerbate conflicts between tourists and residents (Hughes 2018). What’s worse, the COVID-19 pandemic escalates the existing conflicts as residents condemn tourists as carriers of the virus and believe tourists contaminate communities (Los Angeles Times 2020), which hinders the move toward destination sustainability (Wulfhorst 2017). The introduction of the sharing economy sheds light on the current clash by facilitating satisfying resident-tourist relationships and enhancing destination sustainability. The extant literature review indicates a lack of theoretically innovative studies on the resident-tourist relationship and its role in destination sustainability (Font and McCabe 2017). Therefore, this study intended to: (1) review relevant theories that provide the theoretical foundation for the resident-tourist relationship in the sharing economy context; (2) develop a conceptual framework of destination sustainability, and (3) justify destination sustainability from the capital theory approach. It will contribute to the sustainable literature in the sharing economy era and offer guidance in addressing the resident-tourist relationship.

## Literature Review and Theoretical Foundation

Proposed by Gretzel et al. (2020), the impact of COVID-19 pushed research to a crossroads to transform through challenging existing paradigms with a six-pillar approach (historicity, reflexivity, transparency, equity, plurality, and creativity). In this section, the existing literature and selected theories were first reviewed from the “historicity” perspective, extracting linkage among established theories and serving as the theoretical foundation. The study first identifies stakeholders in the sharing economy (Stakeholder Theory), their needs (Collaboration Theory), and resources as bases for exchange (Resource Theory). Then, value co-creation (Service-Dominant Logic) explains stakeholders’ needs and resources to supplement each other. Finally, the rationale of destination sustainability is justified by analyzing four capitals from the Capital Theory Approach.

### Stakeholder Theory

Sharing economy literature on destinations (Boes et al. 2016; Leung et al. 2019) identifies tourists, residents, government, and sharing economy platforms as the four key stakeholders.

### Collaboration Theory

Collaboration theory serves as a useful tool to resolve conflicts and advance shared visions among different stakeholders (Jamal and Getz 1995) by identifying each stakeholder’s needs. Tourists pursue authentic experiences, better value for money, and sustainable tourism products in the sharing economy environment (Cheng 2016). Residents’ needs are summarized as reaping direct economic benefits from tourist activities (Lee 2013). The driving forces behind government involvement include the ardent interest in tourism’s economic returns, the mitigation of the undesirable effects of tourist activities, and the implementation of destination sustainability (Kubickova 2016). Last, sharing economy platforms enable tourism enterprises to expand their scopes, generate platform users, and make profits (Teixeira and Ferreira 2019).

### Resource Theory

Resource theory offers stakeholders guidance to exchange resources they possess to meet their needs and form peer-to-peer collaboration. Time, money, and involvement are tourists’ resources enclosed in their travel experience (Prebensen et al. 2013). The principal resource owned by residents is local authenticity, which consists of genuine quality, originality, uniqueness, sense of place, and pride (Gannon 1994). The government has legislative empowerment and resources to implement tourism (Ruhanen 2013); sharing economy platforms play the primary role in applying information technology in connecting tourists with residents in destinations.

### Service-Dominant Logic

Service-dominant (S-D) logic provides a system-wide perspective of value co-creation through service exchanges among different stakeholders (Vargo and Lusch 2014). Value-creation in tourism is inextricable from tourists’ participation and involvement (Prebensen et al. 2013), such as feedback. Residents contribute to the value co-creation process as both suppliers of authenticity and co-creators to tourists’ experience (Stylidis et al. 2014). The government joins the value co-creation process as a facilitator and regulator (Ruhanen 2013) by regulating unethical and illegal behaviors on tourists’ rights, securing locals’ safety and benefits, and cohering efforts to destination sustainability. During the value co-creation process, sharing economy platforms enable efficient synergies of residents and tourists, and the platform itself profits for a fraction of the sharing fee (Belk 2014).

### Capital Theory Approach to Sustainability

The capital theory approach (CTA) uses all capital assets’ economic value to measure sustainability (Ruta and Hamilton 2007). These capital assets can be categorized into natural, manufactured, human, and social capital (Ekins 1992). Applying CTA to destination sustainability, this study posits that a destination’s total capital should not decline over time for sustainable growth.

## A Conceptual Framework of Destination Sustainability

**Fig. 1.**
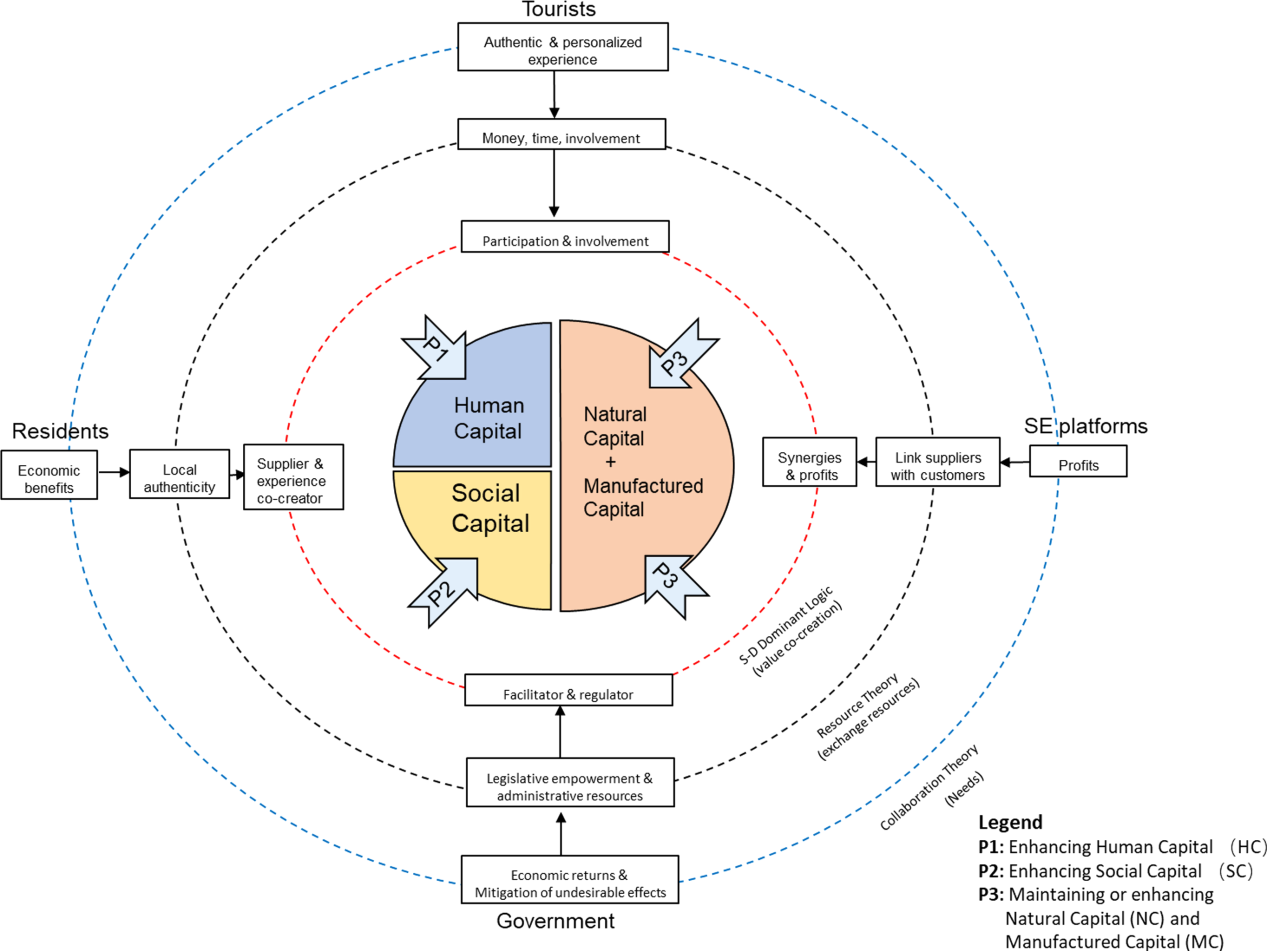
A conceptual framework of destination sustainability built on sharing economy

The relevant literature review provides a solid foundation to connect the related theories and guide tourist destinations’ sustainable development. The proposed framework was aligned with the six-pillar shift advocated in the transformative e-Tourism research (Gretzel et al. 2020). The careful examination (historicity) and reflection (reflexivity) on the current literature generated a different approach (creativity) to the sharing economy’s function in all related stakeholders (equity). Additionally, this framework revealed the sharing economy’s implicit value in destination sustainability (transparency) through building bridges that lead to an alternative to the current challenge in practice (plurality). Figure 1 (below) presents the proposed conceptual framework.

### Resident-Tourist Relationship Facilitated by Sharing Economy

The proposed framework identifies four stakeholders, whose unique needs and possessed recourses are marked in the blue and black dash circles (Fig. 1). The value co-creation process occurs within the red-dash circle of the framework. In short, the new peer-to-peer collaboration paradigm ensures value co-creation in destinations through exchanging resources and meeting all stakeholders’ needs.

### Destination Sustainability Built on Sharing Economy

In the center of the framework (Fig. 1), CTA explains how the multi-stakeholder approach of value co-creation in the sharing economy contributes to destination sustainability. Human capital consists of the health, knowledge, skills, and motivation of residents, tourists, and employees at both sharing economy platforms and government level. Such initiatives enhance the total human capital of the destination. Hence, the following proposition:

#### Proposition 1:

*Peer*-*to*-*peer collaboration in the sharing economy and a multi*-*stakeholder approach to value co*-*creation will enhance destinations’ human capital.*

Further, the government builds trusted, accessible systems of governance, regulations, and justice, while the sharing economy platform provides safe, supportive working environments and intelligent networks. Such peer-to-peer collaboration forms a new paradigm of social relationships that contributes to the increase of social capital. Thus, the following proposition is put forward:

#### Proposition 2:

*Peer*-*to*-*peer collaboration and a multi*-*stakeholder approach to value co*-*creation will enhance the destination’s social capital.*

As the core assets of destinations, natural and cultural resources are well-maintained to attract tourists due to value co-creation in the destination. Therefore, we formulate the following proposition:

#### Proposition 3:

*Peer*-*to*-*peer collaboration and a multi*-*stakeholder approach to value co*-*creation will maintain or enhance the destination’s combined natural capital and manufactured capital.*

The framework indicates that a favorable resident-tourist relationship can only be sustained when all stakeholders have a common interest in keeping this mechanism running. In the proposed context, all the stakeholders contribute their resources to and benefit from the destination development, resulting in an operationally sustainable system.

## Implications

### Theoretical Implications

In response to the recent criticism on lacking macro-level guidance in the literature (Gretzel et al. 2020), the current study makes the first attempt to conceptually explore multi-stakeholder collaboration in the sharing economy. The IT-facilitated sharing economy platform also serves as an active stakeholder and contributor instead of an “instrumental solution” criticized by Gretzel et al. (2020). Moreover, this study contributes to the destination literature by first adopting CTA from the economic discipline to measure destination sustainability in terms of four significant capitals: human, social, natural, and manufactured. This study also expands the original CTA in tourist practices by emphasizing human capital and social capital in destination development.

### Practical Implications

The study provides a roadmap with a multi-stakeholder approach to destination sustainability. Stakeholders involved should create a favorable environment and conditions to generate such collaboration. The study confirms the theoretical possibility of solving the conflicts and urges all stakeholders to rethink tourist activities’ value and reexamine their resources to exchange.

## Limitations and Future Research

This study is not without limitations. It did not consider the different levels of destination development. Further, this study developed a conceptual framework but did not provide empirical evidence. However, real-world practices have shown the development tendency identified in the proposed framework. A collaborative app, *i*-*Tourguide*, was developed in China recently to offer tourists audio guide services. The government encourages and invites residents to contribute their expertise in exchange for economic gains, facilitating local tourism recovery. All identified stakeholders in the framework are geared to co-create value and benefit from the prorated profit-sharing mechanism in a sustainable pattern. Future studies should also consider assessing destination development at various levels when a destination implements the framework and collects either qualitative or quantitative data to validate the model.
